# The nature and intensity of mechanical stimulation drive different dynamics of MRTF-A nuclear redistribution after actin remodeling in myoblasts

**DOI:** 10.1371/journal.pone.0214385

**Published:** 2019-03-28

**Authors:** Lorraine Montel, Athanassia Sotiropoulos, Sylvie Hénon

**Affiliations:** 1 Matière et Systèmes Complexes, CNRS UMR 7057, Université Paris Diderot, Sorbonne Paris Cité, Paris, France; 2 Institut Cochin, INSERM U1016, CNRS UMR 8104, Université Paris Descartes, Sorbonne Paris Cité, Paris, France; University of Toronto, CANADA

## Abstract

Serum response factor and its cofactor myocardin-related transcription factor (MRTF) are key elements of muscle-mass adaptation to workload. The transcription of target genes is activated when MRTF is present in the nucleus. The localization of MRTF is controlled by its binding to G-actin. Thus, the pathway can be mechanically activated through the mechanosensitivity of the actin cytoskeleton. The pathway has been widely investigated from a biochemical point of view, but its mechanical activation and the timescales involved are poorly understood. Here, we applied local and global mechanical cues to myoblasts through two custom-built set-ups, magnetic tweezers and stretchable substrates. Both induced nuclear accumulation of MRTF-A. However, the dynamics of the response varied with the nature and level of mechanical stimulation and correlated with the polymerization of different actin sub-structures. Local repeated force induced local actin polymerization and nuclear accumulation of MRTF-A by 30 minutes, whereas a global static strain induced both rapid (minutes) transient nuclear accumulation, associated with the polymerization of an actin cap above the nucleus, and long-term accumulation, with a global increase in polymerized actin. Conversely, high strain induced actin depolymerization at intermediate times, associated with cytoplasmic MRTF accumulation.

## Introduction

The development and differentiation of cells are known to be influenced by mechanical cues, such as deformation, stress, or the rigidity of the substrate (reviewed in [1]). In particular, changes in the mechanical properties of the surrounding matrix or tissue can alter cellular fate in diseases such as fibrosis, atherosclerosis, and cancer [2]. During development, the interplay between forces and deformation of the tissue organizes the embryo through gastrulation or neural closure [3], highlighting the importance of understanding the mechanics of morphogenesis.

At the cellular level, the influence of mechanical cues have been studied on a broad scale, ranging from that of single proteins, where cryptic sites can be unraveled by traction forces (reviewed in [4]), to the global cellular scale, with the reorganization of the lamellipodium during motion (reviewed in [5]) or the realignment of stress fibers in response to cyclic strain [6,7].

The main component of the mechanical signal converges towards the actin cytoskeleton, as it is the central agent of mechanical organization. Indeed, the mechanical properties of cells, such as their rheology and mechanosensitivity, primarily rely on their cytoskeleton, which is highly dynamic as the result of the constant interplay between cytoskeletal proteins and the mechanical environment. Adhesion proteins, which cross-talk with the actin cytoskeleton, are at the forefront of the mechanosensory apparatus of the cell. At the other end of the mechano-transduction pathways, the activity of transcription factors, such as YAP/TAZ and MRTF/SRF, have been shown to be mechanically dependent (reviewed in [8]).

Serum response factor (SRF) [9] and its cofactors myocardin-related transcription factors (MRTF) [10,11] are actin-sensitive transcription factors that regulate diverse biological functions, such as neural development [12,13], the circadian clock [14], fibroblast to myofibroblast transition [15,16], and muscle differentiation and fusion [17,18]. Together, they control the expression of hundreds of genes, especially those of the cytoskeleton, such as the actins [19]. MRTF family members MRTF-A (MKL1/MAL/BSAC) and MRTF-B (MKL2/MAL16) are highly homologous [10,13,17]; MRTF-A is the most studied one and is often used as a paradigm of the family.

The SRF/MRTF pathway is known as a crucial regulator of muscle homeostasis in response to mechanical cues: it is required to increase muscle mass in response to mechanical overload, and its decreased activity during the lack of mechanical activity (or disuse atrophy) participates in muscle wasting [20,21].

The regulation of SRF activity by MRTF-A is controlled by the localization of MRTF-A in the cell: SRF is located in the nucleus where it can bind DNA, whereas MRTF-A, as a large protein (~145kD), shuttles between the nucleus and the cytoplasm through the Importin αβ and Exportin 1-mediated nuclear transport mechanism [22,23]. However, the bipartite nuclear localization signal (NLS) of MRTF-A is embedded in three G-actin-binding RPEL motifs [24–26]. Thus, the NLS is accessible only when actin is not already bound. Indeed, the availability of actin monomers determines accessibility of the NLS and the intracellular localization of MRTF-A; when monomers are abundant, the NLS is hidden and MRTF-A is sequestrated in the cytoplasm, whereas when they are scarce, MRTF-A can be imported into the nucleus and bind SRF [23,27]. Nuclear actin monomers can also prevent SRF binding, even when MRTF-A accumulates in the nucleus. Thus, the localization of actin and its polymerization state in the nucleus are also of importance: over-expression of the actin-NLS [23] blocks MRTF-A from binding SRF, whereas serum-stimulation induces nuclear actin polymerization through RhoA and mDia [28] and MRTF-A nuclear accumulation, as does nuclear actin expulsion by MICAL-2 [29].

The dependence of SRF/MRTF activity on actin dynamics was originally studied in response to biochemical cues, such as serum [9,22,23] and growth factor stimulation [15,30], which activate actin polymerization through the RhoA pathway and the two effectors ROCK [31,32] and mDia [33,28]. However, any signaling pathway that changes the balance between monomeric and filamentous actin can potentially alter MRTF localization. In particular, actin polymerization pathways regulated by small GTPases have been shown to be activated in response to mechanical cues [31,34]. Changes in the cellular geometric constraints through micropatterning [15] or changes in the rigidity of the substrate [35] can alter the F-/G-actin ratio in fibroblasts and epidermal cells and activate the nuclear localization of MRTF-A and the transcription of SRF target genes. The application of a constant global strain for 24 h also induces the transcription of the SRF targets in vascular smooth muscle cells [32], as does application of a cyclic strain to cardiomyocytes [34]. In fibroblasts, a constant force applied through collagen-coated microbeads activates the two RhoA downstream effectors ROCK [31] and mDia [36], and the target genes are activated within hours. More recently, Iyer *et al*. [37] investigated a very rapid dynamic regime (shorter than 5 min) in live HeLa cells and observed rapid actin polymerization and MRTF-A accumulation upon global force stimulation. All but the last of these studies where performed on fixed samples, with only the last two [31,37] exploring the dynamics of the system and finding very different response times (from 5 to 50 min) for MRTF-A nuclear accumulation and actin polymerization.

Here, we assessed the dynamic responses of actin and MRTF-A to two different types of mechanical stimulation, local or global, over a large range of time scales, from a few minutes to two hours, and investigated the unexplored question of the role of strain level. We used myoblasts, a cell type that has seldom been studied in the context of MRTF-A/SRF, although this pathway is central to the adaptation of skeletal muscle to force. We observed a strong correlation between actin polymerization and relocation of MRTF-A into the nucleus. First, we showed that the location of MRTF-A-GFP within myoblasts correlates with its expression level and the levels of G- and F-actin. Second, we used custom-built magnetic tweezers to show that a force applied through a single bead can trigger the local polymerization of actin around the bead and relocation of MRTF-A to the nucleus within 30 min. Finally, we used a stretching device that applied controlled strains and observed nuclear relocation of MRTF-A under moderate strain at two timescales: a few minutes, and a few tens of minutes. We linked those two regimes to the reorganizations of two different parts of the actin cytoskeleton, apical and basal stress fibers. Under higher strain, we showed that the first rapid response is maintained, though the actin cytoskeleton is later disrupted and MRTF-A relocates to the cytoplasm.

## Materials and methods

### Cell culture

C2C12 murine myoblasts from the ATCC were grown in DMEM with 10% FCS, 1% penicillin, and streptomycin at 37°C and 5% CO2. DMEM with red phenol was replaced by DMEM without red phenol the day before the experiments to allow optimal fluorescence imaging.

### Transfection and live markers

For live staining of F-actin, SiR-actin (Spirochrome) was added to cells at a concentration of 50 nM approximately 15 h before the experiment and used without rinsing. DAPI was added to live cells 30 min before the experiment. The MRTF-A-GFP plasmid has been described previously [23]. The mCherry-actin and LifeAct-mCherry plasmids were kind gifts of Maïté Coppey (Institut Jacques Monod, Paris, France) and Claire Hivroz (Institut Curie, Paris, France). For all experiments, except those with SiR-actin, cells were transfected using Nanofectin (PAA Laboratories, Pasching, Austria). Approximately 110,000 cells were transfected 18 h before the experiment, following the manufacturer’s instructions, with 0.75 to 2.5 μg DNA and incubated for 6 h with 0.75 to 2.5 μg Nanofectin. Co-transfection with mCherry-actin was performed by adding 1 μg mCherry-actin DNA and 1 μg Nanofectin and according to the manufacturer protocol. After the end of commercialization of Nanofectin, transfections were performed using Lipofectamine 3000 (Invitrogen), using the same amount of DNA. According to the manufacturer protocol, Lipofectamine and DNA were incubated with the cells for 24 h and left in the culture medium throughout the experiment.

### Immunostaining

Cells were fixed in a 4% paraformaldehyde solution for 20 min and stained with phalloidin Alexa Fluor 647 or 488 at 0.026 nmol/l, DNase-I Alexa 594 at 0.16 nmol/l and DAPI at 1 μg/ml (all from Life Technologies) for 30 min at room temperature or overnight at 4°C, after permeabilization with 0.5% Triton X-100 in PBS and saturation. Staining of MRTF-A in un-transfected cells was performed with anti-MRTF-A antibody H140 (Santa Cruz Biotechnologies), at 0.8 μg/ml for 30 min at RT, and anti-rabbit Alexa Fluor 488 (Life Technologies) at 4 μg/ml for 30 min at RT.

### Western blot analysis

Cells were lysed directly in 1x Laemmli buffer and proteins were separated through denaturating SDS-PAGE electrophoresis using Mini-Protean TGX precast gels (Biorad) and transferred on Nitrocellulose membrane using the wet method (Biorad). Membranes were blocked with 5% skim milk in TBS-1% Tween (TBST) 1h at room temperature and probed overnight at 4°C with primary antibodies in TBST 2% milk. The following antibodies were used: rabbit anti-MRTF-A (Abcam, ab49311, 1/500), rabbit anti-pan actin (Cytoskeleton, AAN01-A, 1/750) and mouse anti-hsc70 (Santa Cruz, SC7298, 1/1 000). Following washing in TBST, membranes were hybridized with goat anti-mouse and goat anti-rabbit secondary antibodies coupled to HRP (ThermoFisher, 62–6520 and A27036, 1/10 000). Proteins were revealed using SuperSignal West Femto substrate (ThermoFisher). Proteins were quantified by using FusionCapt Advance software (Vilber Lourmat).

### Quantification of F-/G-actin ratio

The ratio of filamentous (F-) to globular (G-) actin was determined using the G-actin/F-actin in vivo Assay Kit (Cytoskeleton, BK037). Briefly, myoblasts were harvested and lysed 10min at 37°C in Lysis and F-actin Stabilization Buffer. Lysates were cleared by centrifugation at 500 g for 5 min. Subsequently, supernatants were centrifuged at 100,000 g for 1 h at 37°C, which resulted in F-actin in the pellet and G-actin in the supernatant. The F-actin containing pellet was resuspended and solubilized in F-actin depolymerization buffer at a volume equal to the G-actin-containing supernatant volume. Equivalent volumes of supernatant and pellet were resolved by SDS-PAGE and subjected to immunoblot analysis using an anti-pan actin antibody (Cytoskeleton BK037). The F-/G-actin ratio was quantified by using FusionCapt Advance software (Vilber Lourmat).

### Magnetic tweezers

The custom built magnetic tweezers [38,39] are based on an electromagnet (66.5 mm long coil of approximately 800 turns of 0.5 mm copper wire, forming 8 layers) placed around a cylindrical soft-iron core (5.10 mm in diameter, 144 mm long) with a 60° cone-shaped tip (see S1A Fig). The electromagnet was powered by a current of intensity up to 1.2 A through a home-made current generator controlled by a function generator (TG1010, TT Instruments). The magnetic tweezers were used to apply local forces to cells through adhesive 4.5-μm super-paramagnetic beads (Dynabeads M450 Epoxy Invitrogen). The force applied to a bead depends on the current provided to the coil and the distance from the bead to the tip. The forces were pre-calibrated by suspending the Dynabeads in liquid polydimethylsiloxane (PDMS) of known viscosity; for each current provided to the electromagnet, the bead velocity *versus* the distance to the tip was measured by analyzing the recorded trajectories the and force was calculated using Stokes law. A force *versus* distance calibration was obtained for each current (see S1B Fig). For magnetic tweezers experiments, the beads were coated with fibronectin (5μg fibronectin for 4.10^7^ beads for 30 min at 37°C), then saturated with 10 μg/mL BSA for 30 min at 37°C. Cells were seeded on 22 x 22 mm glass coverslips coated with fibronectin (5 μg/mL in DMEM for 30 min at 37°C), 24 h before the experiment. Thirty minutes before the experiment, a suspension of fibronectin-coated beads was added to the cells and left to incubate for 30 min. Just before an experiment, the non-attached beads were removed by gentle rinsing, to avoid accidental mechanical stimulation at this step, and then the coverslip was mounted under the microscope for (Olympus IX81 equipped with a 20x long working distance air objective NA = 0.45, LUCPLFLN). The electromagnet and core were mounted on a micro-manipulator (Inject-Man NI2, Eppendorf) at a 45° angle to the microscope stage (S1A Fig). The axis of the core was aligned with the center of the observation zone. All reported experiments were performed at a distance of 280 μm from the bead to the tip. At this distance, the maximum force that could be applied to a single bead, with the maximum 1.2 A current in the electromagnet, was about 1 nN (see S1B Fig).

### Cell stretcher

Stretching experiments were performed using a custom-built device (S2A Fig) that allowed the cells to be visualized under the microscope while stretching them. Twenty-four hours before an experiment 110 000 cells were seeded on a PDMS disk (30 mm in diameter, 0.3 mm thick, PDMS + 10% curing agent from Sylgard Silicon Elastomer) coated with fibronectin (5 μg/mL in DMEM for 30 min at 37°C). The PDMS disk was mounted between two cylinders. The assembly was placed, with the side on which the cells were seeded face down, in a cylindrical tank which contained culture medium supplemented with 1.5% HEPES. The bottom of the vessel consisted of a glass coverslip 30 mm in diameter to allow observation of the cells under an inverted microscope. The PDMS disk was stretched by pushing a cylindrical transparent plastic post and thus the cells seeded on it were also stretched. The distance between the initial position of the PDMS disk and the final position after pushing the post determined the strain imposed on the disk, which was equal to the relative increase in the surface of the stretched area. Calibration using a PDMS disk micro-patterned with fluorescent fibronectin confirmed a uniform radial strain (see S2B Fig). The measured deformation was also in good agreement with the deformation computed using a simple geometric model (see S2C Fig).

For live experiments, the experimental chamber was mounted on the motorized stage (Prior ProScan II) of an inverted microscope (Olympus IX81 equipped with a 20x long working distance air objective NA = 0.45, LUCPLFLN) and enclosed in a thermalized box (The Cube2, Life Imaging). The desired strain was then applied in less than 5 s at the initial time and kept constant over time. During the first 20 min of the experiment, the sample was scanned to locate cells expressing MRTF-A-GFP and their position was marked. Every 5 to 10 min, a new image of each recorded position was taken, making it possible to follow each cell over time. At the end of a live experiment, the sample could be fixed for later labeling and imaging of the final state.

### Microscopy

Fluorescence images were taken with a 20x air objective (long working-distance, NA = 0.45, LUC PLAN FLN) or a 60x oil immersion objective (NA = 1.42, PlanApo N) in an inverted microscope (Olympus IX81), equipped with an Andor Revolution XD spinning disk confocal set-up (laser diodes 405, 488, 561 and 640 nm; band-pass filters 465/30 nm, 512/23 nm, 607/36 nm, 685/40 nm) and an Andor iXon EMCCD camera. To ensure the validity intensity measurements, all images were taken at the same laser power, gain and exposure time throughout the experiments. In the few cases where it led to over-exposition, the exposure time was reduced, and images taken with lower exposure time were re-scaled during data analysis using image metadata accordingly after prior assessment of the linear relationship between exposure time and intensity.

### Classifying the cells according to MRTF-A-GFP localization

**Fig 1:**
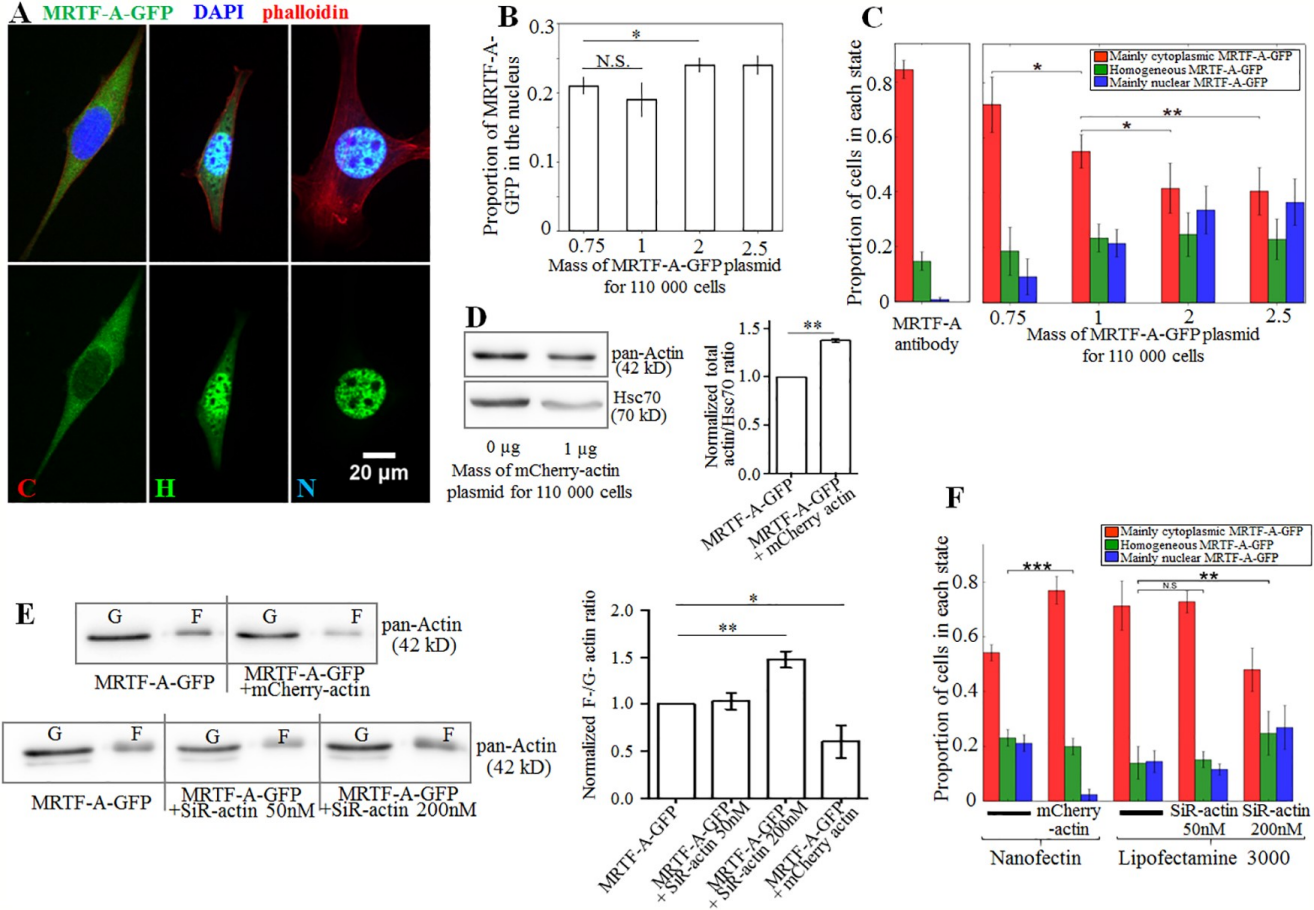
A. Examples of cells classified according to the intracellular localization of MRTF-A GFP, from left to right: mainly cytoplasmic MRTF-A, homogeneously distributed and mainly nuclear MRTF-A. B. Mean fraction of MRTF-A-GFP in the nucleus and C: Cell populations classified by state of main localization of MRTF-A-GFP, as a function of the mass of MRTF-A-GFP plasmid DNA used for transfection with Nanofectine. More DNA led to nuclear accumulation of MRTF-A due to its overexpression relative to that of the G-actin pool (0.75 μg: 75 cells, 1.0 μg: 260 cells, 2.0 μg: 113 cells, 2.5 μg: 126 cells). Cells were grown on PDMS substrates. The reference state for no transfection was obtained using anti-MRTF-A antibody. D. Typical immunoblots for analyzing actin content in C2C12 cells either transfected for MRTF-A-GFP (1 μg of plasmid for 110 000 cells) or co-transfected for MRTF-A-GFP and mCherry-actin (1 μg of each plasmid for 110 000 cells). Hsc70 was used as a loading control; and corresponding quantification of actin expression levels, normalized by Hsc70 (n = 3 independent experiments). E. Typical immunoblots for analyzing the F-/G-actin ratio in C2C12 cells transfected for MRTF-A-GFP (1 μg of plasmid for 110 000 cells or co-transfected for MRTF-A-GFP and mCherry-actin (1 μg of each plasmid for 110 000 cells) or treated with two different amounts of SiRactin, and corresponding quantification of the F-/G-actin ratio (n≥3 independent experiments). With SiR-actin 200nM the F-/G-actin ratio increased by almost 50% with respect to control cells, but does not increase with 50nM SiR-actin; over-expression of mCherry-actin led to a decrease in the F-/G-actin ratio by about 40%. F: Cell populations classified by state of main localization of MRTF-A-GFP as a function of transfection reagent, actin overexpression, and the F-actin stabilizing fluorescent marker SiR-actin: over-expression of actin led to accumulation of MRTF-A-GFP in the cytoplasm whereas SiR-actin 200nM led to accumulation of MRTF-A-GFP in the nucleus.

Cells expressing MRTF-A-GFP were classified according to the major localization of MRTF-A-GFP in the cell, as illustrated in Fig 1A. Cells for which the nucleus was clearly visible and bright in the MRTF-A-GFP channel were labeled as those with mainly nuclear MRTF-A-GFP (“N”, in blue in the graphs). On the contrary, cells for which the nucleus was clearly visible and dark in the green channel were labeled as those with mainly cytoplasmic MRTF-A-GFP (“C”, in red in the graphs). Cells for which the border between the nucleus and cytoplasm could not be distinguished in the green channel were labeled as “Homogeneous MRTF-A-GFP”, or “H” (in green in the graphs). Cells that divided, detached, died or left the image field during an experiment were excluded from all quantitative analyses.

### Quantitative fluorescence analysis

Area, mean intensity, and integrated density of the fluorescence were measured on images with the ImageJ processing program, for two regions of each cell: the whole cell and the nuclear region. The nuclear region was determined by thresholding on the DAPI channel, and the whole cell region was determined by thresholding on the GFP channel of individual cells and drawn by hand when separating adjacent cells was necessary. The proportion of MRTF-A-GFP in the nucleus of a cell was estimated as the ratio of the total intensity in the GFP channel in the nucleus region by the one in the whole cell.

### Database organization and sub-group selection

Data obtained from the experiments were stored in an SQL database to allow data storage for a large number of cells in an easily accessible framework. The parameters of each experiment, such as the date, applied strain level, or starting time of observation, were stored in the Experiment table. Parameters depending on the field of view, such as the total number of cells on the image, were stored in the Zone table. Parameters measured on individual cells, such as plasmid expression, main localization of MRTF-A, or times when MRTF-A localization changed, were stored in the Cell table. Each cell from this table belonged to a field of view identified in the Zone table, and each field of view belonged to an experiment from the Experiment table. This data structure allowed us to select groups of cells following a large variety of queries: for example, all cells expressing actin-mCherry with no more than 25 cells in the field of view, stretched by 10%, for which the initial state was “C” (“mainly cytoplasmic MRTF-A”) and changed state between 10 and 30 min after the start of stretching.

### Statistical analysis

Differences between the numbers of cells in the three categories of MRTF-A-GFP localization were statistically tested, either using a G-test of independence (similar to a chi-square test of independence) or Fisher’s exact test of independence, when the number of cells in one of the categories was too small. Differences between distributions were assessed using a standard t-test. A difference was considered significant when the *p*-value was < 0.05 and the threshold was corrected for multiple comparisons when necessary.

## Results

### The subcellular localization of MRTF-A correlates with its expression level and the F-actin to G-actin ratio

We assessed MRTF-A localization using C2C12 myoblasts transfected with a plasmid encoding MRTF-A-GFP (described in [23]). The subcellular localization of MRTF-A-GFP was observed by fluorescence microscopy and the cells were classified into three categories depending on the localization of MRTF-A-GFP (see Materials and methods): mainly nuclear (“N”), mainly cytoplasmic (“C”), or homogeneously distributed (“H”), as illustrated in Fig 1A. The localization of endogenous MRTF-A in non-transfected cells was assessed through immunostaining: it was mainly cytoplasmic for more than 85% of the cells, as expected from the literature [22] (Fig 1C and S3 Fig). Various amounts of MRTF-A-GFP plasmid were used for transfection, ranging from 0.75 to 2.5μg of DNA for 110,000 cells. We verified that MRTF-A-GFP protein expression paralleled plasmid amounts (S4 Fig). MRTF-A-GFP was mainly cytoplasmic in all transfection conditions, with 18 to 24% of MRTF-A-GFP in the nuclei on average (Fig 1B) and the proportion of MRTF-A-GFP in the nuclei was significantly increased in cells expressing higher amounts of MRTF-A-GFP (Fig 1B). Accordingly there were always more cells in the “C” state (mainly cytoplasmic MRTF-A) than in the “H” and “N” states, and the proportion of C cells for MRTF-A-GFP was always smaller in transfected cells than in non-transfected cells for endogenous MRTF-A (Fig 1C), decreasing from 70% for the lowest concentration of plasmid, down to 40% for the highest. Thus, the more plasmid used for transfection, the more MRTF-A accumulates in the nucleus. This is consistent with known mechanisms for the regulation of intracellular localization of MRTF-A through G-actin-binding. When MRTF-A-GFP is overexpressed due to multiple plasmid copies, there is insufficient G-actin to bind it and maintain it in the cytoplasm. The excess MRTF-A is thus free to accumulate in the nucleus. Even the lowest concentration of plasmid tested here caused an increase in nuclear localization of MRTF-A (Fig 1C). For all subsequent experiments, 1 μg of plasmid was used, as a compromise between sufficient transfection efficiency and low-level overexpression.

Co-transfection with mCherry-actin and MRTF-A-GFP expression vectors increased the levels of G-actin available to bind MRTF-A: the total actin level increased by about 40% (Fig 1D) and the F-actin / G-actin ratio decreased by about 40% (Fig 1E). Meanwhile the localization of MRTF-A-GFP was shifted towards the cytoplasm (Fig 1F). Thus, MRTF-A-GFP is maintained in the cytoplasm when the total amount of G-actin in the cell increases. Finally, the F-actin / G-actin ratio was altered using SiR-actin, a fluorescent probe for F-actin derived from the actin-stabilizing drug jasplakinolide (see Materials and methods). The pool of G-actin was depleted by the stabilizing effect of SiR-actin on the actin filaments when used at a concentration of 200 nM, thus favoring the accumulation of MRTF-A in the nucleus (Fig 1E and 1F). On the contrary SiR-actin had no measurable impact on the F-/G-actin ratio nor on MRTF-A localization when used at concentration 50 nM (Fig 1E and 1F).

### The application of local force induces actin polymerization and accumulation of MRTF-A in the nucleus

**Fig 2:**
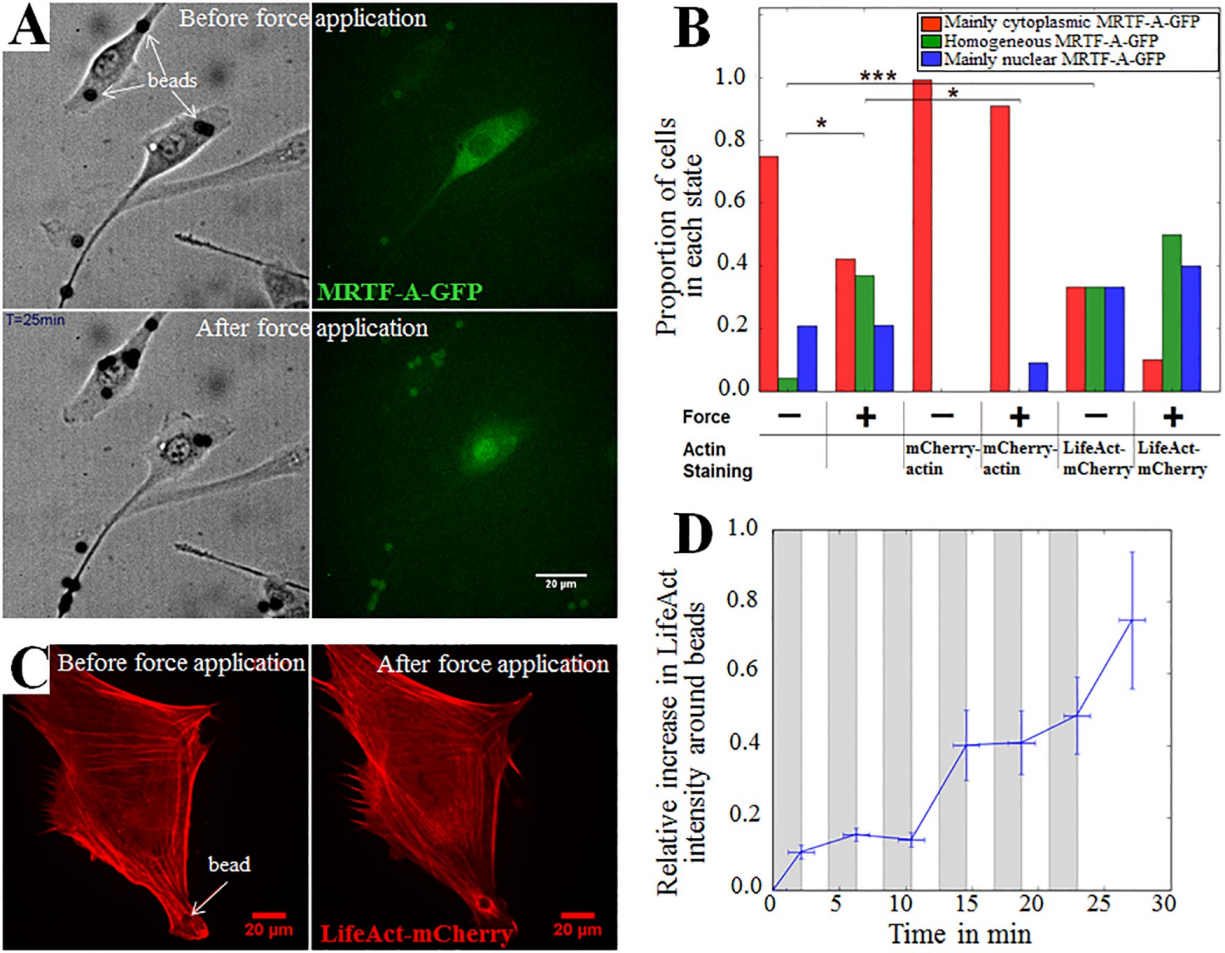
Fibronectin-coated beads actuated by magnetic tweezers induced local actin reorganization and global re-localization of MRTF-A-GFP to the nucleus. A. Live images (20x objective, 0.45 NA) of a cell with adhesive fibronectin-coated microbeads before the application of force, and after six force application and release cycles, each step lasting 125 seconds, for a total of 25 min. The beads appear in black. By the end of the experiment, MRTF-A-GFP relocated to the nucleus. B. Final state of MRTF-A-GFP localization in cells subjected to a 1 nN force through adhesive fibronectin-coated microbeads. The force was applied six times for 125 seconds, and released for the same time, for a total of 25 min. Number of cells: 29 for the control, 23 with force, 4 for control with mCherry-actin, 11 with mCherry-actin and force, 10 for control with LifeAct-mCherry, 6 with LifeAct-mCherry and force. *p* values were calculated using Fisher’s exact test (*p < 0.05, **p < 0.01, ***p < 0.0001). C. Images of the actin cytoskeleton with LifeAct-mCherry before and at the end of the application of force (same experimental conditions as in A, except for objective: 60x, 1.42 NA). An actin ring is clearly visible around the bead at the end of the experiment. D. Relative increase in the mean fluorescence intensity of LifeAct-mCherry per pixel in the vicinity of a bead (see S5 Fig) relative to the fluorescence mean intensity per pixel for the whole cell when subjected to repeated force. Number of cells: 6. The zones in grey correspond to the application of force and the zones in white to the release of force.

We performed the first set of experiments to measure the impact of mechanical cues on the localization of MRTF-A using magnetic tweezers. A constant force step of about 1 nN was applied for 125s and then released for 125s, and the cycle was repeated six times, over a total of 25 min. The localization of MRTF-A-GFP was assessed at the end of each mechanical stimulation (S1 Movie, Fig 2A and 2B). We tested three populations of C2C12 cells: cells expressing MRTF-A-GFP only, cells co-expressing MRTF-A-GFP and mCherry-actin, and cells co-expressing MRTF-A-GFP and LifeAct-mCherry. The localization of MRTF-A-GFP at the end of mechanical stimulation is displayed in Fig 2B for the three cell populations.

In the absence of actin or actin-binding protein overexpression, mechanically stimulated cells (“**+**” in Fig 2B) showed a marked increase in the nuclear localization of MRTF-A-GFP as compared to control non-stimulated cells (cells without beads in the same experimental fields of view, “−” in Fig 2B). This response to force was also associated with a local increase in the amount of F-actin around the mechanically-stimulated beads (S2 Movie and Fig 2C): in cells expressing LifeAct-mCherry, a marker of F-actin, the mean fluorescence intensity per pixel in the red channel was measured in a 5-μm-wide ring-shaped area at the periphery of each bead (see S5 Fig) and normalized to the mean intensity per pixel for the entire cell. This ratio increased by 80% on average throughout the complete force application process (Fig 2C and 2D), evidencing a strong increase in the amount of F-actin in the vicinity of the force application area. Indeed, experiments with optical tweezers on the same cell type have already shown a similar effect [40].

Changing the F/G-actin equilibrium interfered with the nuclear re-localization of MRTF-A-GFP under mechanical stress. Overexpression of mCherry-actin completely abolished the mechanically-induced nuclear accumulation of MRTF-A-GFP, with localization similar to that of the un-stimulated control (Fig 2B), whereas cells expressing LifeAct-mCherry, known to stabilize F-actin [41,42], exhibited enhanced nuclear re-localization of MRTF-A-GFP under mechanical stimulation. They also displayed increased nuclear re-localization of MRTF-A-GFP, even without mechanical stimulation (“−” in Fig 2B). This may be due to the stabilizing effect of LifeAct on actin filaments [41,42]. The presence of LifeAct thus favors the nuclear localization of MRTF-A-GFP, similarly to SiR-actin, another stabilizer of actin filaments, as described in the previous paragraph.

Overall, these experiments showed that the local application of a force on myoblasts triggered nuclear accumulation of MRTF-A-GFP, which correlated with the polymerization of actin around the force application area. Stabilizing actin filaments enhanced this effect, whereas over-expressing actin abolished it. These observations are consistent with the known mechanism of mechanical forces mediated actin assembly through the Rho pathway, promoting MRTF-A nuclear accumulation triggered by a shortage of G-actin in the cytoplasm [31,37].

### Real-time monitoring of MRTF-A-GFP localization dynamics in C2C12 cells in response to global strain

**Fig 3:**
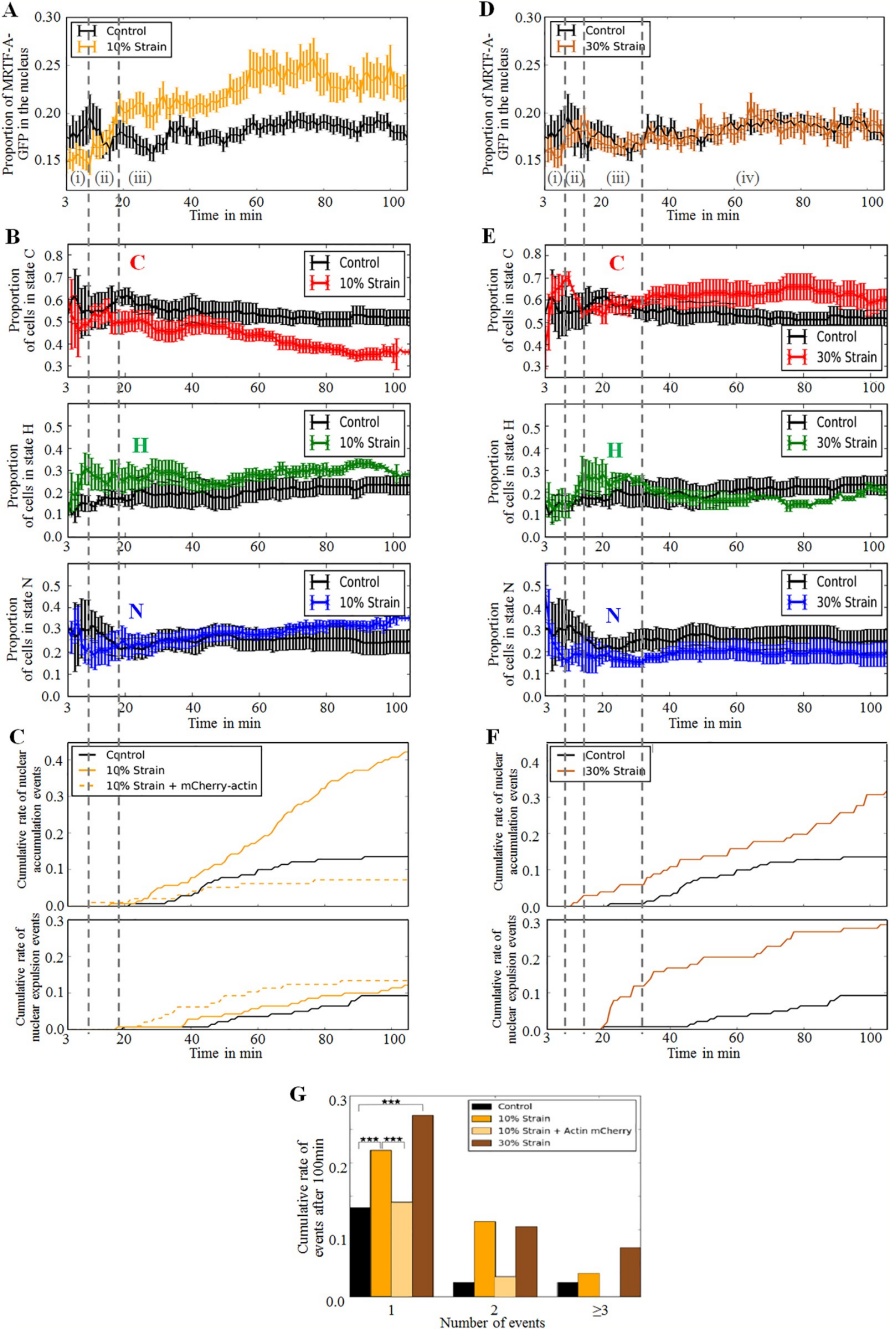
Live monitoring of MRTF-A localization in stretched cells. A. Mean value of the proportion of MRTF-A-GFP in the nucleus of cells as a function of time, in cells stretched by 10% *vs* control (control: 125 cells, n = 5 independent experiments; 10% strain: 109 cells, n = 5 independent experiments). B. Repartitions of the cells among the three categories of MRTF-A-GFP localization: “C” for mainly cytoplasmic, “N” for mainly nuclear, and “H” for homogeneously distributed. The cells were followed over time under microscope (Olympus IX81, 20x long working-distance air objective, NA = 0.45), *t* = 0 corresponds to the beginning of stretching. Cells stretched by 10% *vs* control (control: 143 cells, n = 5 independent experiments; 10% strain: 145 cells, n = 5 independent experiments). Different phases of nuclear accumulation or expulsion of MRTF-A-GFP, (i) to (iv), are described in text. C. Cumulative rates of nuclear accumulation and expulsion events, counted as the number of events per observed cells. A nuclear accumulation event corresponds to the observation of a cell changing from C to H state or from C to N state or from H to N state. A nuclear expulsion event corresponds to the observation of a cell changing from N to H state or from N to C state or from H to C state. D, E, F: same as A, B, C for cells stretched by 30%, *vs* control (control: 125 cells for E, 143 cells for F, n = 5 independent experiments, 30% strain: 100 cells for E, 101 cells for F, n = 3 independent experiments). G. The proportion of cells experiencing 1, 2, or 3 or more changes in main MRTF-A-GFP localization during the 110 min of observations under control (no strain) (143 cells), 10% strain (145 cells expressing MRTF-A-GFP only, 176 cells co-expressing MRTF-A-GFP and mCherry-actin), or 30% strain (101 cells).

In a second series of experiments, cells were seeded onto stretchable fibronectin-coated PDMS disks (see Materials and methods) and cultured for 24 h under standard culture conditions (10% serum). A randomly selected population of cells was then tracked over time: for each experiment, images of areas containing at least one cell expressing MRTF-A-GFP were recorded, with an updated image of each area taken every 5 to 10 min, allowing each cell to be tracked over time. The localization of MRTF-A-GFP in this population was characterized in three complementary ways. First the fraction of MRTF-A-GFP in the nucleus was followed for each individual cell (Fig 3A and 3D). Second the fractions of cells in the three states of major MRTF-A-GFP localization (C for mainly cytoplasmic, H for homogeneously distributed, N for mainly nuclear) were also followed over time (Fig 3B and 3E). Typical images are displayed in S6 Fig and S3 Movie. Although cells were tracked over time, not all cells were observed at the same time. The curves result from several independent experiments, whereas several fields of view were tracked over time during a given experiment. Each point thus gives the state of the cells observed at time *t* +/-3 min after stretching, and the curves start at *t* = 3 min. Finally to gain insight into the dynamics of the cell population, the changes in the state of major MRTF-A-GFP localization in live experiments was also tracked for individual cells. The events were divided into two categories: when the state of a given cell had changed from C to H, or C to N, or H to N (resp. from N to H, or N to C, or H to C) between two consecutive observations, a MRTF-A-GFP nuclear accumulation event (resp. nuclear expulsion event) was counted. The cumulative numbers of events per cell are shown in Fig 3C and 3F. When normalized, the number of events is divided by the number of observed cells.

### The stationary localization of MRTF-A in a population of un-strechted cells is maintained through a dynamic equilibrium

For un-stretched cells the mean localization of MRTF-A-GFP within the cell populations remained roughly stable over time, with a mean nuclear fraction of MRTF-A-GFP of 18% ± 2% (Fig 3A), and with 55% ± 5% of the cells having MRTF-A-GFP mainly in the cytoplasm (Fig 3B). This stability resulted from a dynamic equilibrium, as evidenced in Fig 3C: some cells accumulated MRTF-A-GFP in the nucleus over time, whereas MRTF-A-GFP exited the nucleus for others. The rate of nuclear expulsion events was very low and constant over time, one event for approximately 400 cells and per min. The rate of nuclear accumulation events was slightly more variable, but with the same mean value, and the population state stayed stable over time.

### A moderate strain induces both short- and long-term nuclear accumulation of MRTF-A-GFP

In stretching experiments, PDMS disks seeded with cells were subjected to static strain applied at time *t* = 0 and held constant over time for the duration of the experiment, typically 110 min. Two different levels of strain were tested, a moderate strain, consisting of a 10% increase in the area of the stretchable substrate, and a higher, 30%, strain. In all cases, the behaviors of cells under applied stress were various and a statistics of at least a hundred cells was necessary to recapitulate in a reproducible way the diversity of cells response to applied stress.

For the 10% stretch experiments (Fig 3A and 3B), there were three phases: (i) between *t* = 3min and *t* = 10min after stretching, the proportion of MRTF-A-GFP in the nucleus was smaller than in the control, evidencing a short term nuclear expulsion of MRTF-A-GFP, consistent with the observed decrease in the percentage of N cells in favor of H cells. In a second phase (ii), between *t* = 10min and *t* = 20min, a rapid nuclear accumulation of MRTF-A-GFP was observed, with the mean proportion of MRTF-A-GFP in the nucleus increasing from about 15% to more than 20%. During this second phase, the proportions of cells in each category (C, H, N) changed only slightly, with a slow increase in the proportion of N cells, probably because the cells have not yet accumulated enough MRTF-A-GFP in the nucleus to change categories. Finally, (iii) for *t*>20min, we observed a slower increase in the nuclear proportion of MRTF-A-GFP, which tends to saturate after 80min to about 24% on average, and consistently a progressive decrease (resp. increase) of the number of cells in the C state (resp. N state).

Accumulation (or expulsion) of MRTF-A-GFP was slow: cells went from C to N state (or from N to C) through the intermediate H state in 99% of the observed transitions, which means it took more than 20 min for MRTF-A-GFP to accumulate in the nucleus or in the cytoplasm (10min was the mean interval between two successive observations of the state of the same cell).

The rate of nuclear expulsion events when cells are stretched by 10% was the same as in the control (Fig 3C), low and constant over time, about one event for approximately 400 cells per min. On the contrary, the rate of nuclear accumulation events was about two times higher than in the control, a little higher than one accumulation event for approximately 200 cells per min on average. There was hence a net balance towards nuclear accumulation at all times >20min, which resulted in a decrease in the number of C cells, as observed in Fig 3B.

In summary, a 10% strain triggered an increased nuclear localization of MRTF-A-GFP that was maintained for at least 1.5h.

### A higher, 30%, strain hinders nuclear accumulation of MRTF-A-GFP, leading to an active equilibrium between expulsion and accumulation

At first glance, the MRTF-A nuclear ratio appeared almost as stable for the cells stretched by 30% as for the control experiments (Fig 3D). This could lead to believe that a higher strain abolished the response seen for lower deformation. However, the counting of MRTF-A-GFP localization states and state change events (Fig 3E and 3F) told a very different story: under 30% strain, the percentages of N cells at *t* = 3 min was approximately 40% (Fig 3E), compared to about 25% in the non-stretched state, demonstrating rapid nuclear accumulation of MRTF-A-GFP at very short times. There were then four phases: (i) (*t* < 9 min after stretching) an increase in the number of C cells and a decrease in the number of N cells accompanied by a small decrease in the proportion of MRTF-A-GFP in the nucleus, down to about 15%; (ii) (9 < *t* < 15 min) a decrease in the number of C cells and an increase in the number of H cells, accompanied by a rapid recovery of the nuclear proportion of MRTF-A-GFP up to 18%; (iii) (15 < *t* < 30 min) a second slow increase in the number of C cells, and small decrease in the nuclear proportion of MRTF-A-GFP; finally (iv) (*t* > 35 min), a phase during which the different measured quantities are almost constant.

Hence, in cells stretched by 30%, the mean nuclear fraction of MRTF-A-GFP showed only small variations, but as a result of an intense dynamical equilibrium, as evidenced in Fig 3G: cells stretched by 30% went through nearly as many MRTF-A-GFP nuclear accumulation events as cells stretched by 10%, which occurred earlier than in control and 10% stretching experiments, and they went through even more expulsion events, with a rate of nuclear expulsion events steeply increasing to a value almost 10 times higher than in control experiments at times *t* = 20min-40min. The stability of the MRTF-A-GFP nuclear ratio thus covered an intense cross-over between cells accumulating and expulsing MRTF-A, that almost compensated each other in terms of global mean MRTF-A nuclear ratio.

### Both levels of strain caused a significant increase of re-localization events in the population, which are suppressed by over-expressing actin

There was a clear difference between the total number of change of state events for the control and stretching experiments during the 100 min of an entire experiment (Fig 3G). In control experiments (without strain), less than 20% of the cells experienced one or several changes in the main localization of MRTF-A-GFP during the 100 min. The number of events per cell doubled when a 10% strain was applied for 100 min, with approximately 38% of the cells showing a change in the main localization of MRTF-A-GFP at least once during the experiment. Under a 30% applied strain, this proportion increased to 46%. Strain markedly increased the number of cells undergoing two transitions: from 6% for the control, to 20% for the 10% stretch, and 24% for the 30% stretch.

Overexpression of actin through mCherry-actin completely inhibited nuclear translocation of MRTF-A under mechanical stimulation, as previously observed in the magnetic tweezers experiments: the proportion of cells displaying cytoplasmic localization of MRTF-A-GFP remained constant over time, as in the control experiments, and the number of events was also very similar (Fig 3C and 3G), with MRTF-A retained in the cytoplasm with the increased pool of G-actin.

### A moderate strain induces actin polymerization, whereas a high strain induces actin depolymerization at intermediate times

**Fig 4:**
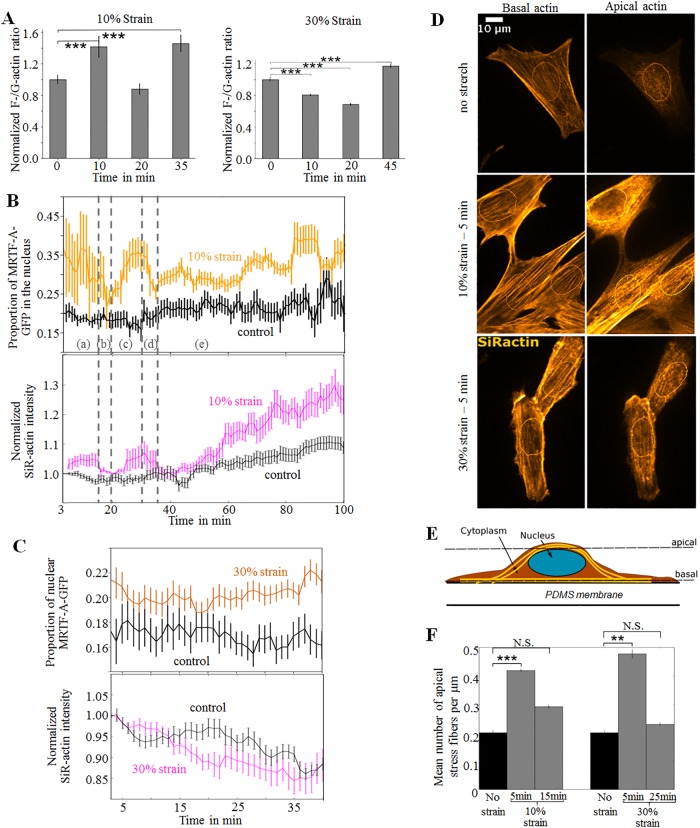
A. F-/G-actin ratio in populations of cells subjected to 10% or 30% strain and stained after fixation at different times of stretching with phalloidin Alexa 647 for F-actin and DNase-I Alexa 594 for G-actin, measured as the ratio between total fluorescence intensities in the far red to red channels. (10% Strain, n = 2 independent experiments, 0min: 214 cells, 10min: 223 cells, 20 min: 246 cells, 35min: 248 cells. 30% Strain, n = 2 independent experiments, 0min: 310 cells, 10min: 331 cells, 20min: 280 cells, 45min: 175 cells). B. Mean value of the proportion of MRTF-A-GFP in the nucleus of cells and median value of the SiR-actin intensity relative to the initial fluorescence level as a function of time, in cells stretched by 10% *vs* control (10% strain: n = 3 independent experiments, 93 cells; control: n = 2 independent experiments, 124 cells). C. Same measurements for cells stretched by 30% vs control (30% strain: *n* = 4 independent experiments; 108 cells. Control *n* = 2, 53 cells). D. Confocal microscopy images of myoblasts in which F-actin was labelled with SiR-actin after fixation. Top: un-stretched control cell; top left: basal layer showing aligned actin stress fibers (z = 0); top right: apical actin with un-organized actin structure (z = 2.25 μm). Middle: cells after 5 min of 10% strain; middle left: basal layer with aligned actin stress fibers (z = 0), middle right: organized actin cap with aligned stress fibers above the nucleus (z = 2 μm). The contour of the nuclei was measured from the DAPI signal and drawn on the images. Bottom: cells after 5 min of 30% strain; bottom left: the cells have partly detached from the stretched substrate (z = 0), bottom right: organized actin cap with aligned stress fibers above the nucleus (z = 1.75 μm). E. Schematic view of a cell, showing the apical and basal levels that were imaged. F. Mean number of stress fibers above the nucleus rescaled by the size of the nucleus (width in μm in the direction perpendicular to the stress fibers). Control: 38 cells; 10% strain—t = 5 min: 66 cells, 10% strain—t = 15 min: 55 cells, 30% strain—t = 5min: 50 cells, 30% strain—t = 25 min: 41 cells.

We subsequently investigated the expected correlation between increase in F-/G-actin ratio under strain and MRTF-A nuclear accumulation. We first stained fixed cells at different times of stretching (Fig 4A), with phalloidin Alexa 647 for F-actin and DNase-I Alexa 594 for G-actin [43], and used for the ratio between total fluorescence intensities in the far red to red channels as a measure of the F-/G-actin ratio in each cell. Typical images are displayed in S7 Fig. In 10% stretching experiments (Fig 4A left panel), we observed first an increase in the mean F-/G-actin ratio, consistent with phase (i) of Fig 3A and 3B. (fast accumulation of MRTF-A in the nucleus), followed by a decrease in the F-/G-actin ratio, consistent with phase (ii) (accumulation of MRTF-A in the cytoplasm) and a longer term increase in the F-/G-actin ratio, consistent with phase (iii) (accumulation of MRTF-A in the nucleus). In the 30% stretching experiments, we observed a decrease in the F-/G-actin ratio at short times (Fig 4A right panel), and a recovery at longer time scales, consistent with phases (ii) and (iv) of Fig 3D and 3E (respectively nuclear expulsion and re-accumulation of MRTF-A-GFP).

To obtain more precise data on correlation, we monitored the dynamics of both F-actin and MRTF-A-GFP localization at the same time. Typical images are displayed in S8 Fig and S4 Movie. We used the live F-actin fluorogenic probe SiR-actin [44] at a concentration of 50nM, a concentration that did not alter the sub-cellular repartition of MRTF-A-GFP (see Fig 1F). SiR-actin was added to the cell medium and stained all the cells. Its fluorescence was much higher when attached to actin filaments than when in solution [44] and it was used without rinsing, allowing the staining of the newly formed actin filaments. Unfortunately, there is no live-staining available for G-actin. Yet since at the timescale of the experiment (<2h), little expression of new actin is expected [45], the level of F-actin is representative of the F-/G-actin ratio.

In the control experiments with SiR-actin (Fig 4B), unstretched cells showed a slight decrease in short-term SiR-actin intensity (along with a slight decrease in MRTF-A-GFP nuclear localization), presumably due to phototoxicity, as well as a small long-term increase (along with a slight increase in MRTF-A-GFP nuclear localization), revealing that SiR-actin, a derivative of jasplakinolide, still acted as a stabilizer of actin filaments, even at the very low concentration used here.

Live SiR-actin monitoring of stretched cells (Fig 4B) revealed 5 phases in both actin polymerization and MRTF-A-GFP localization in response to a 10% strain. At times t<16 min (phase (a)), F-actin and MRTF-A-GFP nuclear localization both had higher levels than in the control. This first phase was followed by several phases (b to e) during which F-actin and MRTF-A-GFP nuclear localization either decreased or increased together. These different phases were consistent with phases (i) to (iv) as described in Fig 3A and 3B, except for timing, and for phase (d) which had no analog in Fig 3A and 3B.

Fig 4B differs from Fig 3A and 3B because the samples differed in two ways: first the transfection agent used (Nanofectin for the samples of Fig 3 and Lipofectamine for the samples of Fig 4B, see Materials and methods) and second the use of SiR-actin for samples of Fig 4, which could explain the observed slower dynamics since SiR-actin is a stabilizer of actin filaments.

Cells were also stretched by 30%, following the same experimental protocol. The cytoskeleton appeared to be incapable of sustaining such a high degree of deformation: SiR-actin intensity started decreasing as soon as 5 min after the start of stretching and continued to decrease for up to 35 min (Fig 4C), suggesting damage to the cytoskeleton. Actin depolymerization correlated with the nuclear expulsion of MRTF-A-GFP for *t*<15min (Fig 4C). F-actin re-polymerized at longer times, *t* > 35min. This is consistent with results obtained on fixed cells (Fig 4A).

Overall, these results show that stretching the cells triggered either time-dependent assembly or disassembly of actin filaments, depending on the strain level, and that F-actin assembly correlated with nuclear accumulation of MRTF-A-GFP, whereas F-actin disassembly correlated with cytoplasmic accumulation of MRTF-A-GFP.

### Stress fibers form a transient actin cap above the nucleus minutes after stretching

Strikingly at very short time after stretching, in 30% strain experiments the proportion of cells with MRTF-A-GFP mainly in the nucleus (N cells) was higher than in control experiments (Fig 3E), and in experiments with SiR-actin, the proportion of MRTF-A-GFP in the nuclei was higher than in control experiments (Fig 4B and 4C). We fixed samples after 5, 15, or 25 min of stretch to gain insight into the phenomena that occur shortly after the initiation of stretch. SiR-actin was used after fixation to stain F-actin and the cells were observed by confocal microscopy. In the control state (no stretching), apical F-actin showed no particular structure in most of the cells (Fig 4D top right). However, some cells showed parallel and organized stress fibers above the nucleus, a structure known as an actin cap [46,47]: 45% of the cells had no actin cap and 25% had only one fiber. After 5 min of stretch by 10% or 30%, nearly all the cells displayed an organized actin cap and the mean number of stress fibers above the nucleus, rescaled by the width of their nuclei in the direction perpendicular to the stress fibers, doubled after 5 min of 10% stretch and more than doubled after 5 min of 30% stretch (Fig 4D middle and bottom right). However, such actin reorganization was highly transient: the actin cap returned to its control configuration after 25 min of maintained stretch. This rapid actin polymerization/depolymerization process correlated with the observed short-term nuclear accumulation/expulsion of MRTF-A-GFP (Figs 3E, 4B and 4C). The overall levels of ventral actin stress fibers did not measurably vary after 5 min of 10% stretch. On the contrary, when cells were stretched by 30%, the ventral stress fibers were damaged (Fig 4D, bottom left) and the overall level of F-actin decreased (Fig 4A and 4C).

Overall, these results show the rapid formation of an actin cap in response to stretch (Fig 4D and 4F), associated with MRTF-A accumulation in the nucleus (Fig 4B and 4C), suggesting that the actin cap could be involved in the rapid cellular response to strain [47].

## Discussion

The actin/SRF/MRTF-A pathway is one of the mechanosensitive signaling pathways, transducing mechanical signals to gene expression, and is at the center of mechanotransduction in muscles. The mechanistic link between the F-/G-actin balance and sub-cellular localization of MRTF-A is well documented [22–27]. The availability of the Nuclear Localization Signal of MRTF-A depends on its binding to monomeric actin, and depleting or increasing the pool of G-actin is sufficient to confine MRTF-A to the nucleus or to the cytoplasm, respectively; mechanical stimulation affects the G-/F-actin ratio, and G-actin level in turn regulates the location of MRTF-A. However, the dynamics of the system are poorly understood.

We conducted studies on C2C12 myoblasts in order to gain insight into the dynamics of MRTF-A relocation after mechanical stimulation. We first confirmed that in the absence of any mechanical stimulation the sub-cellular distribution of MRTF-A closely correlates with the actin F-/G-ratio, with excess G-actin promoting cytoplasmic localization and F-actin stabilization promoting nuclear localization. In addition, we showed that a population of myoblasts under standard culture conditions (*i*.*e*. in a medium containing serum) displays a dynamic steady state for the localization of MRTF-A, in which some cells show nuclear accumulation of MRTF-A, while others show nuclear expulsion, and that the dynamics of MRTF-A shuttle between cytoplasm and nucleus is strongly enhanced under mechanical stress. In response to different types and levels of mechanical stimulation, local through microbeads or global through stretchable substrates, we evidenced a re-location of MRTF-A into the nucleus (resp. into the cytoplasm), correlated to actin polymerization (resp. depolymerization). The time between the observed actin polymerization (or depolymerization) and nuclear (or cytoplasmic) accumulation of MRTF-A was in all cases less than the time resolution of our experiments (a few minutes), as previously observed [40]. We also evidenced, for the first time, a rapid but transient nuclear accumulation of MRTF-A in cells subjected to global stretching, within a few minutes, correlated with the formation of a peri-nuclear actin cap. This is similar to the recent observations of the rapid and reversible assembly of a nuclear actin network in serum-stimulated fibroblasts [28]. However, the F-actin assembly that we observed was peri-nuclear rather than nuclear. Under sustained stretching, we observed long-term actin polymerization, correlated with long-term nuclear accumulation of MRTF-A, lasting for the two hours of our experiments.

It was recently suggested that force transmission from substrate to nucleus by actin stress fibers stretches nuclear pores, reducing their mechanical resistance to molecular transport and increasing nuclear import of YAP [48]. Such a mechanism is expected to play a negligible role for the transport of MRTF-A, a large protein that needs to bind to Importin αβ / Exportin 1 for nuclear import / export, but could play a role for the transport of G-actin.

In conclusion, we have shown that various types of mechanical stimulation on myoblasts induce nuclear accumulation of MRTF-A, which correlates with actin polymerization in various elements of the cytoskeleton. These phenomena occurred over a wide range of time-scales, from the temporal resolution of the experiments (a few minutes) to their duration (approximately two hours). All our results are consistent with known mechanisms of MRTF-A regulation by G-actin, with a strong correlation between F-actin assembly and the nuclear accumulation of MRTF-A. Furthermore, we demonstrated that nuclear accumulation of MRTF-A after mechanical stimulation is maintained over the long-term, which is probably essential for the transcription of genes under the control of SRF/MRTF-A.

Future studies will need to investigate the same questions in more physiological systems, such as primary myoblasts and myotubes.

## Supporting information

S1 Fig A. Schematic view of the magnetic tweezers experiment. The cells are upside down on the top coverslip to obtain the shortest possible distance between the tip of the electromagnet and the attached bead (about 280 μm). B. Examples of calibration curves obtained by measuring the velocity of a bead in a silicone oil with calibrated viscosity under the force of the magnetic tweezers (I = 1.2A in the electromagnet in this example). The applied force is extrapolated to be approximately 1 nN when the bead is at a distance of 280 μm from the tip of the electromagnet.
(TIF)

S2 Fig A. Schematic view of the stretching device. The cells are on the underside of the fibronectin-coated stretched PDMS sheet and are observed from below using an inverted microscope. At time *t* = 0, the transparent post is pushed down to a depth *h*, which causes the strain. B. Images of a PDMS disk micro-patterned with fluorescent fibronectin before (red) and during (cyan) stretch. C. Measured deformation and deformation estimated by a simple geometrical model: Δ*A/A* = (*α*+1) [(1 - *α*)^2^ + *β*^2^]^1/2^ + *α*^2^–1, *α* = *r*/*R*, *β* = *h*/*R*.
(TIF)

S3 Fig Immunofluorescence images of C2C12 cells stained with anti-MRTF-A. MRTF-A is mainly cytoplasmic in almost all cells. 20X air objective.
(TIF)

S4 Fig A. Typical immunoblots for analysing MRTF-A content in C2C12 cells transfected with different quantities of plasmid coding for MRTF-A-GFP (1 μg or 2.5 μg of plasmid for 110 000 cells). Hsc70 was used as a loading control. The level of endogenous MRTF-A is below the sensitivity of the technique, the quantity of MRTF-A-GFP increases with the mass of plasmids. B. Typical images used for quantitative analyses of Fig 1A, MRTF-A-GFP in green, SirActin (200nM) in magenta, DAPI in grey, 20X air objective.
(TIF)

S5 Fig Typical images used for the analyses of magnetic tweezers experiments. See also S1 Movie. A. Cells co-expressing MRTF-A-GFP (in green) and mCherry-actin (in magenta). The expression level of mCherry-actin is very low but sufficient to block MRTF-A-GFP nuclear translocation under applied force. 20X air objective. B. Cells co-expressing MRTF-A-GFP (in green) and actin LifeAct-mCherry (in red). 20X air objective. C. Example of the areas used to assess actin enrichment around the microbead. The mean intensity per pixel in the LifeAct-mCherry channel in a 5-μm wide ring around the bead is compared to that of the whole cell. 60X oil immersion objective.
(TIF)

S6 Fig A typical image of cells expressing MRTF-A-GFP used for quantitative analyses of Fig 3. Cell 1 is in state C, cell 2 in state H and cell 3 in state N. Cells, such as 3, which are not entirely in the field of view were excluded for the measurement of the nuclear proportion of MRTF-A-GFP but used for the classification of main localization of MRTF-A-GFP. 20X air objective. See also S3 and S4 Movies.
(TIF)

S7 Fig Typical images of cells used for the measurement of the F-/G-actin ratio (Fig 4A). Cells were stretched by 30% for 0, 10, 20, 45 min (from left to right), fixed and stained with with phalloidin Alexa 647 for F-actin (yellow, top) and DNase-I Alexa 594 for G-actin (cyan, bottom). 20X air objective. Scale bars: 50 μm.
(TIF)

S8 Fig Typical images used for live measurement of the MRTF-A-GFP nuclear fraction and SiR-actin intensity (Fig 4B). All the cells are stained with SiR-actin (in magenta) and DAPI (in grey), but only some of them express MRTF-A-GFP (in green). 20X air objective, scale bar: 50μm.
(TIF)

S1 Movie Live monitoring of a cell with magnetic beads subjected to a force thanks to magnetic tweezers (same cell as in Fig 2A). MRTF-A-GFP progressively accumulates in the nucleus. 20X air objective.
(AVI)

S2 Movie Live monitoring of a cell with a magnetic bead subjected to a force thanks to magnetic tweezers (same cell as in Fig 2C). The intensity of LifeAct-mCherry, progressively increases in the vicinity of the bead, evidencing in increase in F-actin content. 60X oil immersion objective.
(AVI)

S3 Movie A cell strained by 30% followed in time, counted from the application of stretch. Its state of main MRTF-A-GFP localization changes several times, from N at 11min to H at 25min, C at 32min, back to H at 96min and to weakly C at 110min.
(AVI)

S4 Movie Live monitoring of mCherry-actin (in magenta) and MRTF-A-GFP (in green) in a 10% stretching experiment. The cells are also stained with DAPI (in blue). The cell in the middle of the first image undergoes a mitosis and is excluded from analyses, as well as its two daughter cells. 20X air objective.
(AVI)

S5 Movie Live monitoring of F-actin (stained with SiR-actin 50nM, in magenta) and MRTF-A-GFP (in green) in a 10% stretching experiment. The cell on the left is the only one in the field of view expressing MRTF-A-GFP. During the course of the experiment, MRTF-A-GFP accumulates in the nucleus and actin polymerizes, as evidenced by the increase of SiR-actin intensity. 20X air objective.
(AVI)
