# Supplementary figures and images for: The nature and intensity of mechanical stimulation drive different dynamics of MRTF-A nuclear redistribution after actin remodeling in myoblasts
Source: PLoS One. 2019 Mar 28;14(3):e0214385. doi: 10.1371/journal.pone.0214385 (PMC6438519; doi:10.1371/journal.pone.0214385)

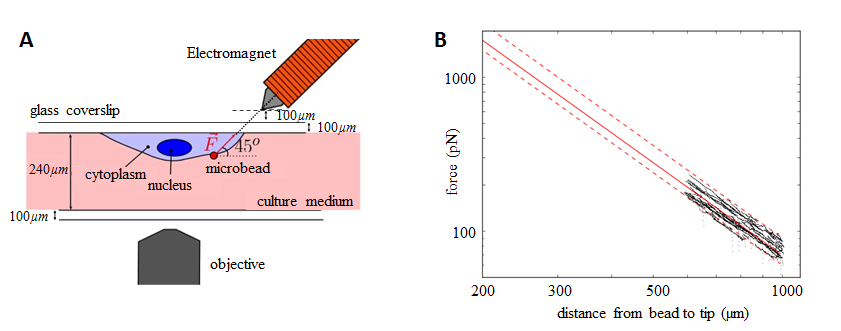

Supplement: S1 Fig — A. Schematic view of the magnetic tweezers experiment. The cells are upside down on the top coverslip to obtain the shortest possible distance between the tip of the electromagnet and the attached bead (about 280 μm). B. Examples of calibration curves obtained by measuring the velocity of a bead in a silicone oil with calibrated viscosity under the force of the magnetic tweezers (I = 1.2A in the electromagnet in this example). The applied force is extrapolated to be approximately 1 nN when the bead is at a distance of 280 μm from the tip of the electromagnet. (TIF) [file pone.0214385.s001.tif]

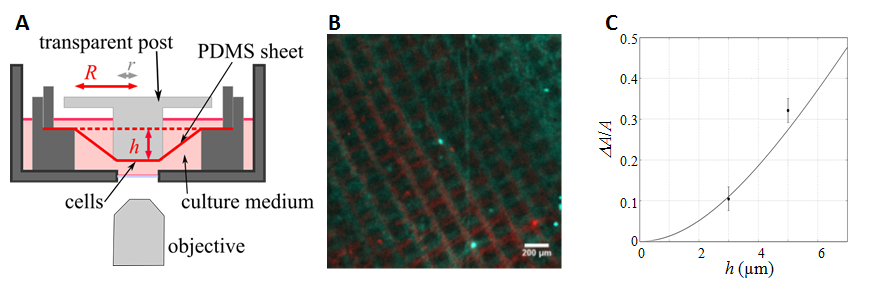

Supplement: S2 Fig — A. Schematic view of the stretching device. The cells are on the underside of the fibronectin-coated stretched PDMS sheet and are observed from below using an inverted microscope. At time t = 0, the transparent post is pushed down to a depth h, which causes the strain. B. Images of a PDMS disk micro-patterned with fluorescent fibronectin before (red) and during (cyan) stretch. C. Measured deformation and deformation estimated by a simple geometrical model: ΔA/A = (α+1) [(1 - α)2 + β2]1/2 + α2–1, α = r/R, β = h/R. (TIF) [file pone.0214385.s002.tif]

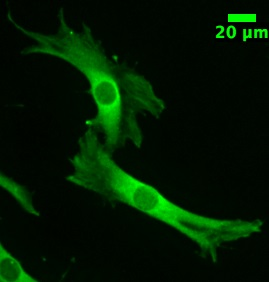

Supplement: S3 Fig — MRTF-A is mainly cytoplasmic in almost all cells. 20X air objective. (TIF) [file pone.0214385.s003.tif]

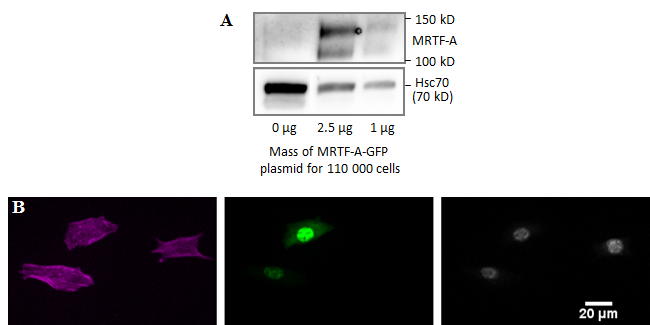

Supplement: S4 Fig — A. Typical immunoblots for analysing MRTF-A content in C2C12 cells transfected with different quantities of plasmid coding for MRTF-A-GFP (1 μg or 2.5 μg of plasmid for 110 000 cells). Hsc70 was used as a loading control. The level of endogenous MRTF-A is below the sensitivity of the technique, the quantity of MRTF-A-GFP increases with the mass of plasmids. B. Typical images used for quantitative analyses of Fig 1A, MRTF-A-GFP in green, SirActin (200nM) in magenta, DAPI in grey, 20X air objective. (TIF) [file pone.0214385.s004.tif]

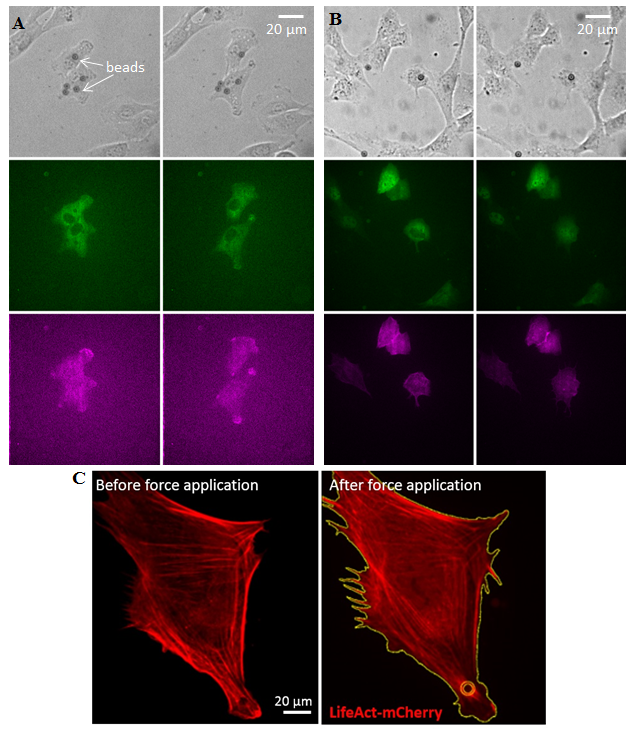

Supplement: S5 Fig — See also S1 Movie. A. Cells co-expressing MRTF-A-GFP (in green) and mCherry-actin (in magenta). The expression level of mCherry-actin is very low but sufficient to block MRTF-A-GFP nuclear translocation under applied force. 20X air objective. B. Cells co-expressing MRTF-A-GFP (in green) and actin LifeAct-mCherry (in red). 20X air objective. C. Example of the areas used to assess actin enrichment around the microbead. The mean intensity per pixel in the LifeAct-mCherry channel in a 5-μm wide ring around the bead is compared to that of the whole cell. 60X oil immersion objective. (TIF) [file pone.0214385.s005.tif]

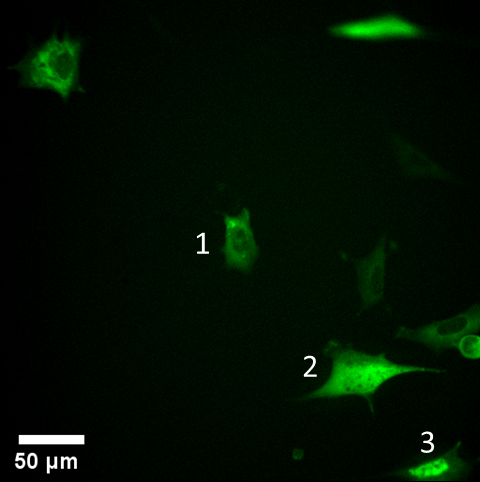

Supplement: S6 Fig — Cell 1 is in state C, cell 2 in state H and cell 3 in state N. Cells, such as 3, which are not entirely in the field of view were excluded for the measurement of the nuclear proportion of MRTF-A-GFP but used for the classification of main localization of MRTF-A-GFP. 20X air objective. See also S3 and S4 Movies. (TIF) [file pone.0214385.s006.tif]

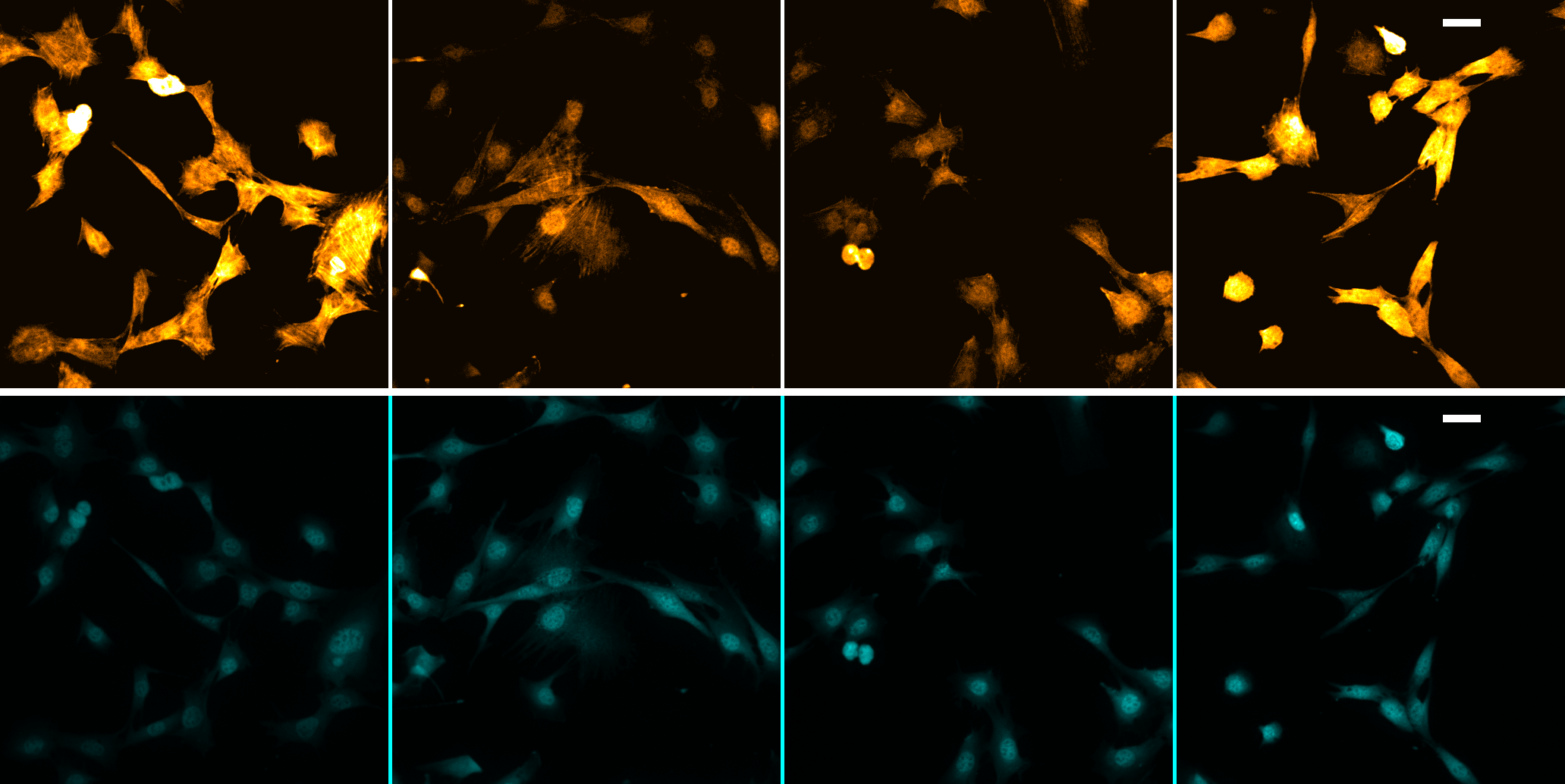

Supplement: S7 Fig — Cells were stretched by 30% for 0, 10, 20, 45 min (from left to right), fixed and stained with with phalloidin Alexa 647 for F-actin (yellow, top) and DNase-I Alexa 594 for G-actin (cyan, bottom). 20X air objective. Scale bars: 50 μm. (TIF) [file pone.0214385.s007.tif]

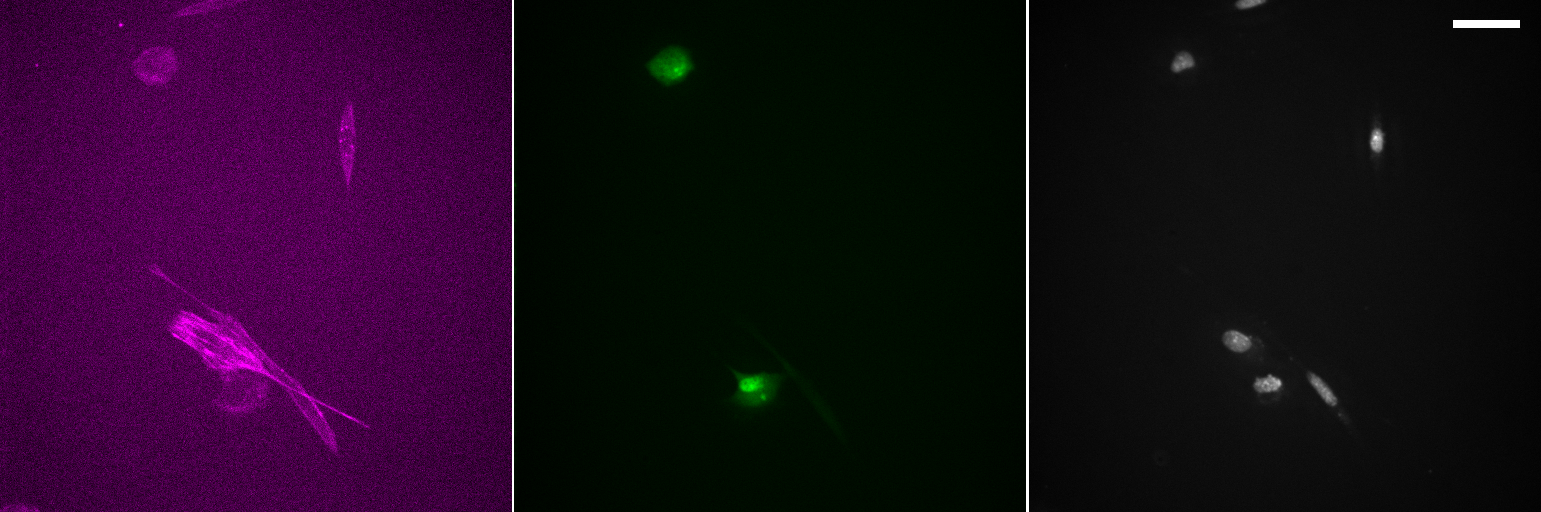

Supplement: S8 Fig — All the cells are stained with SiR-actin (in magenta) and DAPI (in grey), but only some of them express MRTF-A-GFP (in green). 20X air objective, scale bar: 50μm. (TIF) [file pone.0214385.s008.tif]
